# Cognitive and Structural Correlates of Conversational Speech Timing in Mild Cognitive Impairment and Mild-to-Moderate Alzheimer’s Disease: Relevance for Early Detection Approaches

**DOI:** 10.3389/fnagi.2021.637404

**Published:** 2021-04-27

**Authors:** Céline De Looze, Amir Dehsarvi, Lisa Crosby, Aisling Vourdanou, Robert F. Coen, Brian A. Lawlor, Richard B. Reilly

**Affiliations:** ^1^Trinity Centre for Biomedical Engineering, School of Engineering, Trinity College Dublin, Dublin, Ireland; ^2^Mercer’s Institute for Successful Ageing, St James’s Hospital, Dublin, Ireland; ^3^Institute of Neuroscience, School of Medicine, Trinity College Dublin, Dublin, Ireland

**Keywords:** speech timing, conversation, cognitive function, brain volumes, Alzheimer

## Abstract

**Background**: Increasing efforts have focused on the establishment of novel biomarkers for the early detection of Alzheimer’s disease (AD) and prediction of Mild Cognitive Impairment (MCI)-to-AD conversion. Behavioral changes over the course of healthy ageing, at disease onset and during disease progression, have been recently put forward as promising markers for the detection of MCI and AD. The present study examines whether the temporal characteristics of speech in a collaborative referencing task are associated with cognitive function and the volumes of brain regions involved in speech production and known to be reduced in MCI and AD pathology. We then explore the discriminative ability of the temporal speech measures for the classification of MCI and AD.

**Method**: Individuals with MCI, mild-to-moderate AD and healthy controls (HCs) underwent a structural MRI scan and a battery of neuropsychological tests. They also engaged in a collaborative referencing task with a caregiver. The associations between the conversational speech timing features, cognitive function (domain-specific) and regional brain volumes were examined by means of linear mixed-effect modeling. Genetic programming was used to explore the discriminative ability of the conversational speech features.

**Results**: MCI and mild-to-moderate AD are characterized by a general slowness of speech, attributed to slower speech rate and slower turn-taking in conversational settings. The speech characteristics appear to be reflective of episodic, lexico-semantic, executive functioning and visuospatial deficits and underlying volume reductions in frontal, temporal and cerebellar areas.

**Conclusion**: The implementation of conversational speech timing-based technologies in clinical and community settings may provide additional markers for the early detection of cognitive deficits and structural changes associated with MCI and AD.

## Introduction

### Rationale and Research Goals

Progressive loss of cognitive function and progressive cerebral atrophy are characteristic features of Mild Cognitive Impairment (MCI) and Alzheimer’s disease (AD; Dubois et al., 2007; McKhann et al., 2011; Kirova et al., 2015; König et al., 2015; Szatloczki et al., 2015). Early and cost-effective diagnosis is crucial for the development and establishment of early interventions and to make effective treatment decisions.

Increasing efforts have focused on the establishment of novel biomarkers for the early detection of AD and prediction of MCI-to-AD conversion, including clinical, brain, genetic, and neuropsychological data. Behavioral changes over the course of healthy ageing, at disease onset and during disease progression, have been recently put forward as promising markers for the detection of MCI and AD. Repeated behavioral measures taken from everyday situations (e.g., walking speed) and/or extracted from tests that can be easily implemented outside clinical settings may offer the opportunity to increase timely detection and represent additional sources to the standard brain imaging and clinical neuropsychological assessments (e.g., MMSE—Mini Mental State Examination).

Speech-based approaches have proved to perform well in the discrimination of MCI and AD (König et al., 2015; López-de-Ipiña et al., 2015; Weiner et al., 2016; De Looze et al., 2018; Mirheidari et al., 2020). Speech and language impairments are indeed salient characteristics of MCI and early AD (Ripich et al., 1991; Caramelli et al., 1998; Chapman et al., 2002; Carlomagno et al., 2005; Taler and Phillips, 2008; Laws et al., 2010; Gayraud et al., 2011; Ahmed et al., 2013). However, the cognitive and structural underpinnings of these speech-based measures in classification approaches have not been systematically investigated and are not fully established. Understanding these underpinnings could add significant clinical value and further support the potential use and implementation of speech-based technologies in and outside clinical settings for the monitoring of cognitive trajectories.

One candidate tool is the analysis of spontaneous speech in conversational interactions. Engaging in a conversation is a complex skill which requires the integration of multiple independent cognitive subsystems, themselves supported by extensive networks of several brain regions. If one of these subsystems or networks is impaired, conversational speech difficulties may arise. Conversational speech characteristics may therefore be sensitive markers of underlying cognitive and structural impairments. The present study examines whether the temporal organization of speech in a collaborative referencing task is associated with cognitive function and the volumes of brain regions involved in speech production and known to be reduced in MCI and AD pathology. We then explore the discriminative ability of the temporal speech measures for the classification of MCI and AD.

### Speech-Based Approaches for the Early Detection of MCI and AD

The potential use of speech-based approaches for the early detection of MCI and AD represents an important line of research in AD speech pathology. Deficits in the lexical, semantic, executive, discourse and pragmatic domains of language are commonly observed in MCI and early AD (Ripich et al., 1991; Caramelli et al., 1998; Chapman et al., 2002; Carlomagno et al., 2005; Feyereisen et al., 2007; Taler and Phillips, 2008; Laws et al., 2010; Gayraud et al., 2011; Ahmed et al., 2013; Drummond et al., 2015; Mueller et al., 2018). Symptoms include word-finding difficulties, decreased semantic and phonemic fluency, lexical richness, syntactic complexity and topic coherence. They often occur before clinical diagnosis and progress over the course of the disease (Ahmed et al., 2017). The articulatory aspects of language production are generally preserved until the late stages of the disease (Croot et al., 2000). Several speech and language tests and measures have been employed for the classification of MCI and AD, with accuracy rates spanning from 0.71 to 0.80 for the discrimination of MCI vs. healthy controls (HCs) and 0.80–0.98 for the AD vs. HC contrast (Roark et al., 2011; Jarrold et al., 2014; Meilán et al., 2014; König et al., 2015; López-de-Ipiña et al., 2015; Dodge et al., 2015; Asgari et al., 2017; Tóth et al., 2018; Gosztolya et al., 2019; O’Malley et al., 2020).

### Speech Timing in Conversational Speech: Cognitive Underpinnings

Engaging in a conversation is a complex skill which requires the integration and coordination of multiple independent cognitive processes as speakers perform a number of tasks simultaneously. They must comprehend their interlocutor’s utterances while, at the same time, prepare their response, keep track of the conversation topic, of the interlocutor’s intent, and anticipate turns ending (Sacks et al., 1978; Riest et al., 2015). Smoothed exchanges of turns rely on the good functioning of a number of different cognitive abilities, including lexical and semantic retrieval, episodic memory, sustained attention, working memory, executive function, and language comprehension (Mueller et al., 2018).

Deficits in every one of these domains and conversational speech and language difficulties have been documented in adults with MCI and AD (Carlomagno et al., 2005; Feyereisen et al., 2007; Taler and Phillips, 2008; Rousseaux et al., 2010; Forbes-McKay et al., 2013; Drummond et al., 2015; Fraser et al., 2016). Difficulties in understanding words and sentences and producing words have been attributed to impairments in lexical and semantic retrieval (Murdoch et al., 1987; Forbes-McKay et al., 2013). Difficulties in discourse organization and turn-taking management are thought to stem from deficits in executive functioning (Rousseaux et al., 2010; Ash et al., 2012).

The temporal aspects of conversational speech within the frame of turn-taking organization may be a particularly sensitive marker of an individual’s cognitive capacity. Analyses of connected speech revealed that AD speech is characterized by slower speech rate (global speed of speech including pauses), a higher number of silent pauses, longer pauses and shorter interpausal units (or chunks of speech bounded by silent pauses; Weiner et al., 2008; Davis and Maclagan, 2009; Rousseaux et al., 2010; Hoffmann et al., 2010; Gayraud et al., 2011; Pistono et al., 2016; De Looze et al., 2018). Slower speech rate, a higher number of silent pauses, a reduction in phrase length and an increase in speech turns frequency were also observed in MCI and AD conversational speech (Carlomagno et al., 2005; Hoffmann et al., 2010; Sajjadi et al., 2012).

Slower speech rate, larger pause frequency, and longer pause duration have been mainly attributed to lexico-semantic deficits in MCI and AD (Goldman Eisler, 1968; Hoffmann et al., 2010; Forbes-McKay et al., 2013; Pistono et al., 2016). Other studies have also pointed towards further deficits in working memory, attention, and executive function (Ash et al., 2012; Pistono et al., 2016; De Looze et al., 2018). Longer pauses between clauses have been associated with speech planning difficulties (Matsumoto et al., 2013). In addition, the manner in which readers chunks their speech stream into units of different sizes was shown to be dependent on their working memory (WM) capacity. In healthy older adults, readers with low WM capacity were more likely to chunk their speech into smaller units than those with high WM, indicating a narrower scope of planning (Ferreira and Swets, 2002; Swets et al., 2014). In a previous study, we found that, in overt sentence reading, a higher number of pauses, shorter interpausal units and slower speech rate were associated with reduced language and working memory/attention scores and that these temporal speech characteristics were reflective of difficulties in planning longer and more syntactically complex utterances in healthy older adults and individuals with MCI and AD (De Looze et al., 2018).

Together these separate findings suggest that the temporal organisation of speech in MCI and AD may be indicative of a number of underlying cognitive deficits, e.g., deficits in episodic memory, lexical retrieval, executive functions, working memory and attention. However, these associations are not well established within the frame of conversational interactions.

### Speech Timing in Conversational Speech: Structural Correlates

During conversational interactions, several brain regions are recruited and formed into extensive networks to support visual, phonological, lexical, semantic, syntactic, pragmatic, discourse, and attentional processes.

Besides a limited number of studies describing the neural correlates of conversational speech production, a number of regions are thought to be involved in these cognitive processes. A widespread distribution of language areas in the temporal, parietal, and frontal lobes have been associated with lexical-semantic memory and retrieval (Binder et al., 2009). Naming performance has been associated with the left anterior temporal lobe, including the left temporal pole, the left inferior temporal gyrus (ITG), the left middle temporal gyrus (MTG), the left superior temporal gyrus (STG), and the left fusiform gyrus (L FFG; Kircher et al., 2004; Brambati et al., 2006; Binder et al., 2009; Baldo et al., 2013; Pravatà et al., 2016; Leyton et al., 2019). Involvement of the left inferior parietal gyrus (Kircher et al., 2004; Baldo and Dronkers, 2006) and the left inferior frontal gyrus (IFG; Binder et al., 2009; Hurley et al., 2015) has also been reported. The temporal regions are thought to be related to the activation and storage of lexical representations while the frontal areas have been linked specifically to the retrieval aspect of lexico-semantic processing (Hagoort, 2005; Binder et al., 2009).

Language areas associated with speech planning, executive functions and, more specifically, the monitoring of turn-taking organization, include the motor cortex, the middle and inferior frontal gyri, the inferior parietal lobule (IPL), and the STG (Hagoort, 2005; Matsumoto et al., 2013; Magyari et al., 2014; Foti and Roberts, 2016; Nissim et al., 2017). The left IFG is thought to support the parsing and planning of sentence and discourse-level linguistic information (Matsumoto et al., 2013; Magyari et al., 2014). The midfrontal areas have been related to verbal action planning and attentional control (Hagoort, 2005) and the IPL has been linked to verbal working memory capacity (Deschamps et al., 2014). These regions together are thought to play a central role in sentence and discourse level comprehension processes and control, particularly in turn-ending anticipation (Magyari et al., 2014). Other regions reported to be associated with working memory and executive function in speech processing and production include the precuneus/posterior cingulate cortex and the cerebellum (Cereb; Xu et al., 2005; Hampson et al., 2006; Newman et al., 2013; Bourguignon, 2014; Christodoulou et al., 2014; Hirshorn et al., 2014; Helder et al., 2017). Increased activation of these two regions together with the IFG, MTG, and IPL have been related to working memory capacity in sentence reading and comprehension, potentially reflecting the additional working memory demand that emerges at the sentential/discourse level (Xu et al., 2005; Prat et al., 2007; Newman et al., 2013; Helder et al., 2017; De Looze et al., 2018).

Pauses within clauses, reflective of lexico-semantic processes, have been associated with activation in the superior and middle temporal gyri bilaterally (Kircher et al., 2004). Between-clause pauses, reflective of speech planning and monitoring, have been related to the left STG, the left insula, and the right IFG (Kircher et al., 2004; Matsumoto et al., 2013). Inter-speaker gaps (i.e., the silence between two speakers’ turns), underlying the anticipation of a speaker’s response, have been associated with the posterior temporal gyrus, the supramarginal gyrus, the premotor cortex and middle prefrontal cortex (Bögels et al., 2015; Foti and Roberts, 2016). Speech rate, reflective of speech motor control and planning, has been related to the STG bilaterally, the left MTG, the right ITG, the right fusiform gyrus (R FFG), the left and right IPL, and the precuneus (Ash et al., 2012).

Widespread changes in the structure, function, and organization of a multitude of brain regions have been reported in MCI and AD. Beyond a typical atrophy of the medial temporal lobe (Lehéricy et al., 1994; Chan et al., 2001; Dickerson et al., 2001; Killiany et al., 2002), volume reductions in the fusiform gyrus (FFG), posterior cingulate/precuneus, superior temporal, inferior parietal, and orbito-frontal cortices were also observed in MCI and AD (Tondelli et al., 2012; Wang et al., 2015; Dicks et al., 2018; Verfaillie et al., 2018). Given the overlap of regions engaged in speech processing and production and reduced in MCI and AD pathology, it may be hypothesized that conversational speech timing characteristics may be reflective of underlying regional volume reductions. Evidence in the context of conversational interactions is however limited.

### Objectives and Hypotheses

In this study, we first examine whether the temporal organization of conversational speech in a collaborative referencing task is associated with cognitive function in individuals with MCI and AD. In a second analysis, we investigate whether conversational speech timing is reflective of the underlying volume of brain regions involved in speech production and known to be reduced in MCI and AD pathology. We consider an extensive ensemble of conversational speech timing measures, cognitive domains and brain regions. We expected shorter interpausal units, shorter turns, longer pauses, longer gaps, shorter transition overlaps, a higher number of pauses and gaps and slower speech rate to be associated with lower cognitive function and reduced regional brain volumes. These analyses aim to establish which conversational speech measures reflect underlying cognitive deficits and regional brain volume reductions in order to estimate their clinical relevance for the implementation of speech-based technologies for the monitoring of speech changes in healthy ageing, MCI and AD. Finally, we explore the discriminative ability of these temporal speech measures for the classification of MCI and AD using Cartesian genetic programming (CGP). Although our analyses are exploratory due to the sample size under investigation, to our knowledge, this is the first attempt to examine the discriminative ability of conversational speech measures while also investigating their cognitive and structural underpinnings using the same cohort.

## Materials and Methods

### Ethics Statement

Ethical approval for the study was obtained from the St. James’s Hospital Ethics and Medical Research Committee. Signed informed consent was obtained from all respondents prior to participation.

### Participants

Twenty older adults with MCI and 20 older adults with mild-to-moderate AD were recruited from the Memory Clinic of the Mercers Institute for Successful Ageing (MISA) in St. James’s Hospital, Dublin, Ireland. Forty healthy volunteers (HC) were recruited from the Memory Research Unit in Trinity College Dublin. Participants included in this study were over 50 years of age, fluent in English and literate, to ensure that they could complete all assessments and tasks (*N* = 80). MCI and mild-to-moderate AD diagnoses were based on NIA-AA criteria (Albert et al., 2011; Sperling et al., 2011). Exclusion criteria for healthy participants included history of neurological disorders and/or history of major psychiatric disorders or depression. Thirteen participants with MCI, 13 with AD and 16 HC, without prior MRI contraindications, e.g., pacemakers, cerebral aneurysm clips or other, were randomly selected to undergo brain MRI (*N* = 42). Three participants with MCI, three with AD and four HC were excluded from analysis due to incomplete MRI scans, technical issues with the MRI data (e.g., motion artefact, volume segmentation errors), technical issues with the speech data (e.g., recording issues) and/or abnormal scans or cognitive scores for the HC. Participants with AD, MCI and HC who had reliable cognitive, speech and MRI volumetric measures were included for analysis, resulting in a final sample of 32 individuals.

#### Neuropsychological Tests

All participants underwent two neuropsychological tests, which were administered and assessed by an experienced nurse. The RBANS (Repeatable Battery for the Assessment of Neuropsychological Status; Randolph et al., 1998) includes five cognitive domains: verbal memory (immediate and delayed recall), visuospatial/constructional abilities (figure copy and orientation), attention (symbol and digit coding), working memory (forward and backward digit span) and language (naming and semantic fluency). The MoCA (Montreal Cognitive Assessment; Nasreddine et al., 2005) is composed of 14 tests subsumed under six different cognitive domains which include visuoconstructional/executive function skills (figure copy, clock drawing, and trail test), verbal memory (delayed recall), attention/working memory (sustained attention, serial 7s, forward and backward digit span), language (naming, sentence repetition and phonemic fluency), conceptual thinking (verbal abstraction), and orientation (time and place). Composite scores of five different cognitive domains were computed from age, gender, and education-corrected RBANS and MOCA raw scores, by averaging them for each specific cognitive domain as previously described (De Looze et al., 2018): *Memory* was generated from RBANS and MOCA immediate and delayed recall scores; *Language* from RBANS/MOCA Language and MOCA naming scores; *Working Memory/Attention* from RBANS/MOCA working memory and attention scores; *Visuoconstructional/Executive function* from the RBANS Visuospatial/Constructional scores and MOCA Visuoconstructional/Executive function scores; *Orientation* from MOCA orientation index. All cognitive tests took place in the Memory Clinic of the MISA in St. James’s Hospital. The tests took on an average 35–55 min to complete.

#### Collaborative Referential Task

Participants were asked to engage in a collaborative referential task (Feyereisen et al., 2007; Duff et al., 2013) with a communication partner. The communication partner was the caregiver of the participants with MCI/AD. Each caregiver engaged twice in the referential task, once with the participants with MCI/AD and once with a matched HC. Each pair of individuals engaged in three trials.

In the first trial (*Describe*-Trial), individuals with AD, MCI or the HCs were the directors and the caregivers were the matchers. The directors were given a board with 10 numbered spaces and a set of 10 cards displaying Chinese tangrams arranged on the board in a unique sequence. The matchers were given an identical board with 10 numbered spaces and an identical set of cards which were randomly displayed around the board. The tangrams were black and white geometric shapes which could resemble human beings, animals, or objects but which had no established names. The directors were asked to describe the shapes and tell the matcher where to place them on their board so that, at the end of the trial, the director’s and the matcher’s boards looked alike. In the second trial (*Match*-Trial), the roles were inversed. In the third trial (*Describe and Match*-Trial 3), the pair had to discuss together the identical shapes that they were given and agree on where to place them on their respective boards, so that at the end of the trial their boards matched.

The sets of Chinese tangrams were different for each trial, but the same sets were used across pairs of individuals. During the task, the pairs were seating at a table facing each other. A partial barrier or stand up obscured the view of the other’s board, facial expressions and gestures to rely on speech communication only. The task was presented as a game and the participants were told to have fun. The experimenter was siting aside in the room working on a computer while the pairs played the game. Feedback about the total number of correct card placements was provided after each trial.

All sessions took place in a clinical room located in the Memory Clinic and were audio-recorded. H4n Zoom recorders were used for the recordings. The audio signal was recorded at 44 kHz/16 Bit resolution. The collaborative referential task (three trials) took on average 12 min.

#### Speech Annotation and Measure Extraction

The conversational speech data was annotated using the Praat software (Boersma and Weenink, 2016). Speech units and silences were first automatically determined, using a binary voice activity detection (VAD) algorithm proposed in Sohn et al. (1999). Turns, interpausal units, pauses, gaps, and transition overlaps were then automatically derived from the binary VAD using Praat scripts. Interpausal units are speech units separated by a pause. A turn is defined herein as a unit of speech composed of one or several consecutive interpausal units produced by the same speaker. Pauses are silences within a speaker’s turn. The pause threshold used in the automatic procedure was set at 100 ms to ensure its distinction with silent plosives (Sanderman and Collier, 1995). Gaps are silences at turn boundaries, that is when there is a change in speaker. Transition overlaps denote chunks of speech when two speakers speak simultaneously at turn boundaries. Syllables were automatically aligned to the signal using a modified version of de Jong and Wempe’s (2009) Praat script. The acoustic annotation was manually checked by a speech expert and corrected where needed.

Speech timing measures were automatically extracted using Praat scripts and included *each* participant (AD/MCI/HC)’s total number of pauses; gap/transition overlap ratio; duration of pauses, gaps, transition overlaps, interpausal units, and turns; and speech rate (number of syllables per second including pauses). The number of pauses were normalized to the speaker’s turn. The gap/transition overlap ratio was calculated as the number of gaps divided by the number of overlaps. The higher the ratio, the higher the tendency for an individual to use a gap (rather than a transition overlap) when taking a turn.

#### MRI Protocol and T1w Acquisition

Participants were scanned at the National Centre for Advanced Medical Imaging (CAMI), St. James’ Hospital, Dublin, using a 3T Philip’s Achieva system and 32-channel head coil. A 3D Magnetization Prepared Rapid Gradient Echo (MP-RAGE) sequence was used to acquire various scans in addition to a T1-weighted MR image. Scans included the subsequent parameters: FOV (mm): 240 × 218 × 162; 0.9 mm isotropic resolution; SENSE factor: 2; TR: 2 ms; TE: 2.8 ms; flip angle: 8°. The MRI data was obtained within one to 3 weeks after the cognitive and speech assessments.

#### MRI Data Inspection

FreeSurfer software version 6.0 (Dale et al., 1999) was used to analyze the T1w images with the associated cross-sectional pipeline to derive Regions of Interest (ROIs) in each subject’s native space, using the Destrieux atlas (Destrieux et al., 2010). The technical details of FreeSurfer procedures have been described elsewhere (Dale et al., 1999; Fischl et al., 2002; Han et al., 2006; Jovicich et al., 2006). All unprocessed input volumes were inspected for evidence of motion artefact. Surface segmentation failures were identified using Freeview.

#### Feature Extraction

We selected nine regions of interest (ROIs) which were found to be involved in speech production (Xu et al., 2005; Hampson et al., 2006; Newman et al., 2013; Bourguignon, 2014; Christodoulou et al., 2014; Hirshorn et al., 2014; Helder et al., 2017) and reduced in MCI and AD pathology (Tondelli et al., 2012; Wang et al., 2015; Dicks et al., 2018; Verfaillie et al., 2018): the IFG (the sum of the pars opercularis, pars triangularis and pars orbitalis), the Middle Frontal Gyrus (MFG), the Precuneus (Prec), the IPL (the sum of the Angular Gyrus and the Supra Marginal Gyrus), the ITG, the MTG, the planum temporale in the STG, the FFG, and the Cereb. The volumes of these regions were extracted from FreeSurfer cortical segmentation statistical output. Measures were obtained separately for each hemisphere, which resulted in a total of 18 ROIs. Total Gray Matter volume was extracted to assess Group differences. Estimated Total Intracranial Volume served to control for individual differences in head size in regression analyses.

#### Statistical Analyses

All statistical analyses were performed using R software version 3.6.0 (R Foundation for Statistical Computing, Vienna, Austria; R Core Team, 2015).

##### Data Descriptives

The observed sample was first characterized per Group (HC, MCI, and AD). Continuous variables were described as the mean with standard deviation; categorical variables were given as percentage. Ordinary least square and generalized models, when appropriate, were used for comparison of demographics, neuropsychological scores and speech task competence. HC was set as the reference level.

##### Speech Timing by Group

The effects of Group on the temporal characteristics of speech were assessed through a mixed model approach (Bates et al., 2014). Linear mixed effects models are an extension of simpler linear models. They include both fixed and random effects as predictor variables. They are robust for the analysis of repeated measures designs and can account for both within- and between-subject factors (Littell et al., 1996).

Ten temporal characteristics of speech (i.e., the number of pauses, gap/transition overlap ratio, the duration of pauses, gaps, transition overlaps, interpausal units, and turns, and speech rate) were entered as *dependent variables* (repeated measures) in separate linear mixed-effect models. The dependent variables were log-transformed when appropriate. For all models, *fixed effects* were the Group (AD, MCI, and HC) and Trial (*Describe*-Trial, *Match*-Trial, and *Describe and Match*-Trial), with an interaction term. HC Group and *Describe and Match* -Trial were set as the reference levels. Trial was included as a fixed effect to reflect the participant’s role, hence the different cognitive load and speech task involved in each trial. Speakers constituted the *random intercepts*. All our models were adjusted for age, sex, and education.

The significance of interaction terms was assessed through likelihood ratio tests comparing additive models with models with an interaction term. The significance level was set at *α* = 0.006 to correct for multiple comparison (*α* = 0.05/8 models). Following standard procedures for mixed models (Nakagawa and Schielzeth, 2013), both marginal (*R*^2^m, describing the proportion of variance explained by the fixed factors alone) and conditional (*R*^2^c, describing the proportion of variance explained by both the fixed and random factors) *R*^2^ were computed to assess effect size.

##### Association Between Speech Timing and Composite Scores

The association between the temporal characteristics of speech and the composite scores were assessed using linear mixed effects models. As per above, the number of pauses, gaps and transition overlaps (normalized), the gap/transition overlap ratio, the duration of pauses, gaps, transition overlaps, interpausal units, and turns and speech rate were entered as *dependent* variables (repeated measures) in separate models. The dependent variables were log-transformed when appropriate.

In each model, the five composite scores Working Memory/Attention, Language, Memory, Visuoconstructional/Executive Function and Orientation (continuous variables) and Trial (three levels: *Describe*-Trial, *Match*-Trial and *Describe and Match*-Trial) were entered as *fixed effects*, with an interaction term. *Describe and Match*-Trial was set as the reference level. A stepwise procedure (backward and forward) was employed to assess the significance of the predictors. The composite scores were centered around their mean to reduce multicollinearity.

In all models, speakers constituted the *random intercepts*. All our models were adjusted for age, sex, and education. The significance level in the full models was set at α = 0.006 to correct for multiple comparison (*α* = 0.05/8 models). Marginal (*R*^2^m) and conditional (*R*^2^c) *R*^2^ were used to estimate effect size.

##### Association Between Speech Time and Regional Volumes

A biologically informed ROI-based approach was chosen to explore the association between the temporal characteristics of speech and regional volumes through linear mixed-effect modelling (Bates et al., 2014). The number of pauses, gaps and transition overlaps (normalized), the gap/transition overlap ratio, the duration of pauses, gaps, transition overlaps, interpausal units, and turns and speech rate were entered as *dependent* variables (repeated measures) in separate models. The dependent variables were log-transformed when appropriate.

In each model, the ROIs (z-score transformed) and Trial were entered as *fixed effects*, with an interaction term. ROIs of the left and the right hemispheres were run separately. *Describe and Match*-Trial was set as the reference level. A stepwise procedure (backward and forward) was employed to assess the significance of the predictors. Speakers constituted the *random intercepts*. All our models were adjusted for age, sex, and education. The significance level in the full models was set at *α* = 0.003 to correct for multiple comparison (*α* = 0.05/16 models). Marginal (*R*^2^m) and conditional (*R*^2^c) *R*^2^ were used to estimate effect size.

##### Classification of MCI and AD Based on Speech Features

Finally, we explored the discriminative ability of temporal speech characteristics and the use of CGP, a subtype of Evolutionary Algorithms (EAs), for the classification of MCI and AD.

**Figure 1:**
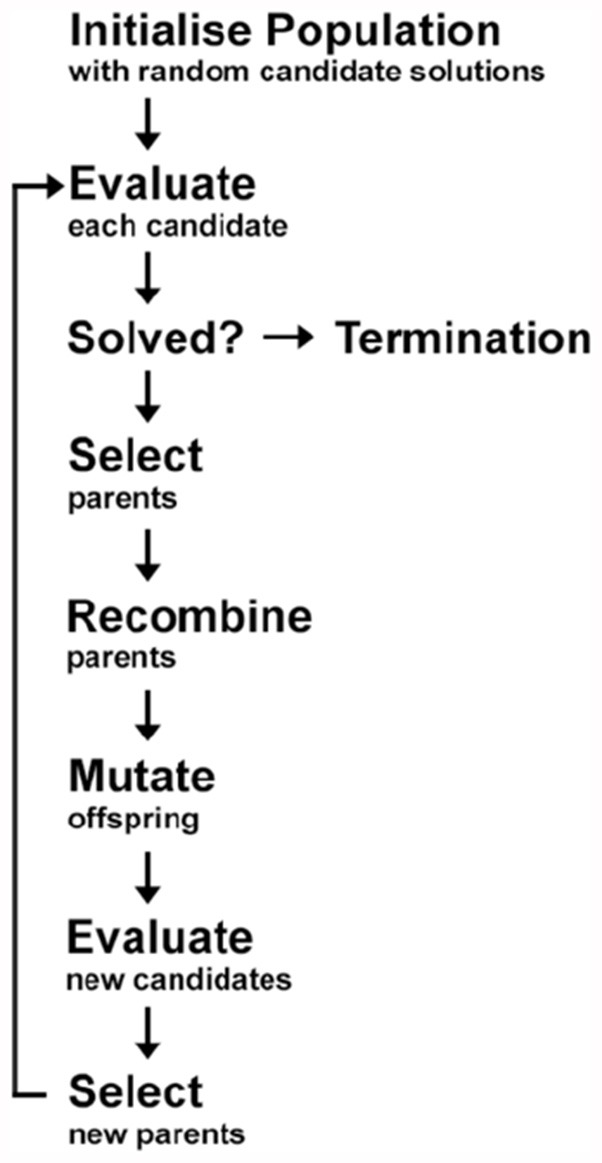
General diagram of the classification process in Evolutionary Algorithms (EAs). In order to find the optimal model (or candidate), a set of working models are randomly generated (Step 1: initialize population). The models (or candidates) are then evaluated to assess their accuracy rate (Step 2: evaluate). In order to achieve the maximum accuracy rate, certain models (or candidates) are selected for use in the subsequent generation of models (Step 3: select) *via* recombination (also known as sexual reproduction or crossover) and/or mutation. Recombination is an operator that is applied to two or more selected models (the so-called parents or genotype or chromosomes), by mixing their genetic material (genes), to create one or more new models (the children or new chromosomes or offspring). Mutation is applied to one model (asexual reproduction) or two models (sexual reproduction) and results in one new model. This procedure is repeated for many iterations and the resulting model is evaluated each time (Step 4: evaluate) or until the desired accuracy rate is achieved at which stage a final optimal model is selected and the process is terminated (Step 5: termination). Adapted from Figure 4.5 of Dehsarvi (2018).

***Rationale for Using Cartesian Genetic Programming.*** EAs are learning algorithms derived from Darwinian evolutionary theory. CGP is a subtype of EAs, which generates directed acyclic computational configurations of nodes. Like other types of EAs, it uses trees as its solution representation (Miller, 2020). CGP can evolve symbolic expressions, Boolean logic circuits, and artificial neural networks. The algorithms generate a population of classifiers through a repeated process of variation and selection. Selection is based on improving fitness criteria when categorizing the participant groups from each other. EAs are stochastic (i.e., different solutions are found each time the algorithms are executed), hence, in order to address this, the best performing classifier was selected from numerous repeated runs of the algorithms. Unlike other standard mathematical approaches and most machine learning algorithms, CGP, like other EAs, makes very few assumptions about the function that generated the data, which allows a wide exploration of the space possible solutions to the problem. The general scheme of an EA is presented in Figure 1.

Computational methods, such as EAs, have been recently used for the measurement and analysis of clinical data (e.g., patient movements data and neuroimaging, among others; Dehsarvi and Smith, 2018). A core advantage when applying EAs with an expressive dynamical representation is that multiple classifiers can be examined. In addition, EAs offer a *white-box solution* for the classification, which is not the case for most (*black-box*) machine learning algorithms. With *white-box* models, the classification process is transparent; it is possible to retrieve how predictions were produced and which variables influenced the population and selection of classifiers. Upon the completion of the classification process, EAs allows for looking into the classification graphs generated by the algorithm and, for instance, exploring how specific features have been chosen to evolve the models. Finally, EAs have proved to perform well with relatively small datasets (Picardi et al., 2017; Dehsarvi and Smith, 2018; Muhamed et al., 2018). To our knowledge, our study is the first to investigate whether the use of EAs and conversational speech may enhance the classification of MCI and/or AD.

**Figure 2:**
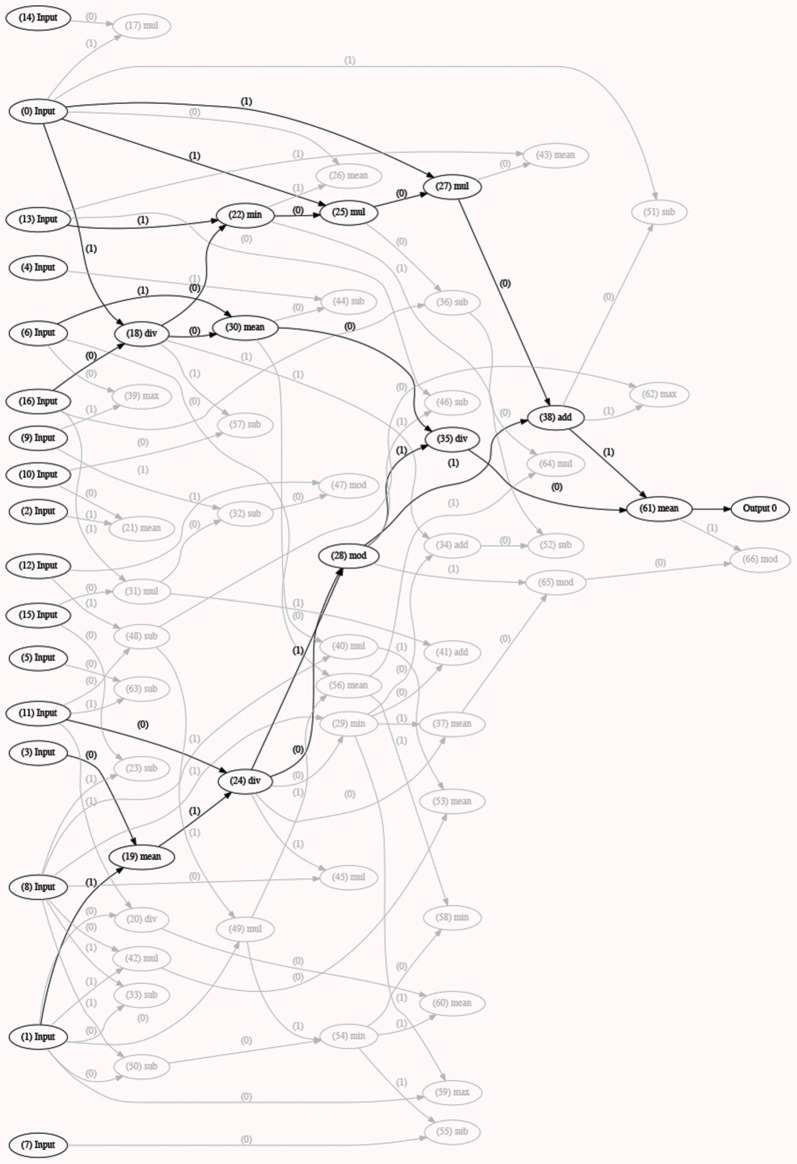
Example of a generation of classification with the optimal model (best fitted chromosome) selected (in black). This model has used a certain number/set of inputs or speech features (inputs 0, 13, 6, 16, 11, 3, and 1) and a combination of different functions to form the best model (or fittest chromosome). Other models with lower accuracy rates are depicted in light gray in the figure. The selected model (or chromosome) is the fittest one of a certain run and is stored as an output, along with all the other runs, upon completion of *5*-fold cross-validation.

***Classification*.** Classification analyses were performed using a novel open source cross platform CGP library (version 2.4; Turner and Miller, 2015). The number of pauses, gaps and transition overlaps, the gap/transition overlap ratio, the duration of pauses, gaps, transition overlaps, interpausal units and turns, and speech rate were used as input features. Per-speaker means and standard deviations of each normalized feature were computed for the three trials separately. Two-class (binary) classification was performed for the AD-HC and MCI-HC contrasts as well as multi-class classification of the three groups. To have equal class representation, the data from each class was randomly divided into subsets of 60% (training), 20% (validation), and 20% (test). The geometry of the programs in the population (referred to as chromosomes) has fifty nodes with a function set of four mathematical operations (+, −, ×, /), multiple inputs (according to the dataset), and one (either class 1 or class 0 for each binary combination of speaker groups) or multiple (one combination per speaker group) outputs. At each generation of classification, the fittest chromosome is selected, and the next generation is formed with its mutated versions (mutation rate = 0.1). Evolution stops when 15,000 iterations are reached. To obtain statistical significance, we completed the analysis for 10 runs for each combination of inputs and the result was calculated as the average of the accuracy rates over the runs. The results (the winning chromosome—an example is provided in Figure 2, the networks, and the accuracy values) were stored for each run individually.

***Five-fold Cross-validation.*** A 5-fold cross-validation was then performed in 10 runs for each combination of inputs to evaluate accuracy and obtain statistical significance. The accuracy was averaged over the runs. An advantage of cross-validation is the production of independent test sets that increases reliability. With *5*-fold cross-validation, one (of *5*) subset is the test set, one subset is the validation set, and the other three subsets are training sets. These sets are alternated, so every set is used once for testing the data. One cycle of the *5*-fold cross-validation does not generate enough classification accuracies to enable comparison, hence, in *5-fold* cross-validation, this is repeated 10 independent times and mean accuracy across all the trials is calculated (with the data samples being randomly allocated in different sets). The results (the winning chromosome, the networks, and the accuracy values) were stored for each run individually and the test results over all the iterations were averaged and reported (Table 6).

**Table 1:** Comparison of demographic and neuropsychological characteristics of participants with mild cognitive impairment (MCI), participants with mild-to-moderate Alzheimer’s disease (AD) and healthy controls (HCs).

				AD vs. HC (*p*-value)	MCI vs. HC (*p*-value)
**Demographics**
Female, %	50	30	42	0.6	0.6
Age, mean (sd)	71.8 (6.9)	74.0 (8.1)	69.8 (6.5)	0.50	0.20
Education, mean (sd)	13.3 (2.5)	13.4 (1.6)	13.2 (1.9)	0.90	0.90
**Clinical characteristics**
RBANS.T, mean (sd)	64.3 (11.8)	84.9 (10.1)	107.6 (12.7)	**0.00**	**0.00**
MOCA.T, mean (sd)	16.8 (4.3)	22.7 (2.5)	27.0 (1.7)	**0.00**	**0.04**
Memory, mean (sd)	14.3 (7.7)	29.3 (6.7)	52.3 (6.9)	**0.00**	**0.00**
Language, mean (sd)	33.8 (15.0)	43.0 (13.1)	51.0 (6.1)	**0.00**	0.12
WM/Attention, mean (sd)	23.8 (18.6)	38.5 (11.5)	50.4 (8.1)	**0.00**	**0.00**
Visuoconstructional/EF, mean (sd)	18.3 (26.0)	43.3 (19.3)	49.1 (8.0)	**0.00**	**0.04**
Orientation, mean (sd)	-20.0 (51.1)	39.0 (17.1)	47.7 (16.4)	**0.00**	0.41
**Structural characteristics**
Total gray matter (cm^3^), mean (sd)	554.3 (55.5)	587.6 (37.2)	581.0 (59.2)	**0.05**	0.34

*Significant differences (*p* < 0.05) between groups are highlighted in bold. RBANS.T, RBANS total score; MOCA.T, MOCA total score; WM, working memory; EF, executive function*.

**Table 2:** Mean and standard deviation of the speech characteristics per group and trial.

Speech measures	*Describe*-trial			*Match*-trial			*Describe and match*-trial		
Mean (sd)	AD	MCI	HC	AD	MCI	HC	AD	MCI	HC
*N* pauses	0.48 (0.17)	0.50 (0.14)	0.42 (0.17)	0.35 (0.10)	0.27 (0.12)	0.33 (0.14)	0.36 (0.16)	0.44 (0.11)	0.37 (0.08)
Gap/overlap ratio	10.35 (7.17)	8.88 (2.85)	6.30 (4.16)	10.43 (7.02)	9.42 (6.67)	5.25 (2.98)	6.12 (3.36)	8.95 (4.94)	5.43 (2.29)
Pause duration	0.94 (1.38)	0.82 (1.28)	0.60 (0.85)	0.85 (1.30)	1.01 (1.53)	0.78 (1.34)	0.64 (0.98)	0.92 (1.38)	0.66 (0.96)
Gap duration	1.13 (1.45)	1.04 (1.55)	0.69 (0.96)	1.36 (2.07)	1.18 (1.83)	0.78 (1.43)	1.23 (1.96)	1.00 (1.67)	0.69 (1.31)
Tov duration	0.17 (0.12)	0.24 (0.18)	0.34 (0.28)	0.22 (0.17)	0.25 (0.20)	0.29 (0.18	0.27 (0.23)	0.23 (0.20)	0.25 (0.19)
IPU duration	0.75 (0.56)	1.05 (0.76)	1.03 (0.80)	0.65 (0.52)	0.71 (0.54)	0.89 (0.67)	0.81 (0.65)	0.97 (0.74)	0.97 (0.68)
Turn duration	3.01 (3.78)	5.20 (5.96)	3.12 (3.68)	1.41 (1.82)	1.48 (2.16)	1.89 (3.04)	2.74 (4.44)	3.19 (4.77)	2.63 (4.31)
Speech rate	3.88 (1.76)	3.81 (1.61)	4.27 (1.93)	4.28 (2.07)	4.16 (2.03)	3.84 (2.09)	4.42 (1.73)	3.99 (1.51)	3.99 (2.03)

*MCI, mild cognitive impairment; AD, Alzheimer’s disease; HC, healthy controls; *Describe*-Trial: individuals with AD/MCI and HC are the directors, i.e., they describe the shapes and instruct where to place them; *Match*-Trial: individuals with AD/MCI and HC are the matchers, i.e., they place the pictures on their board following the caregiver’s instructions; *Describe and Match-*Trial: both interlocutors describe the shapes and agree on where to place them. N, number of; tov, transition overlaps; IPU, interpausal unit*.

**Table 3:** Coefficients and 95% confidence intervals with marginal and conditional *R*^2^ for the observed significant differences in speech features between groups (*p* < 0.006).

Groups	Speech features	Speech features * trials		*R*^2^m; *R*^2^c
	Speech rate	Speech rate * trial	

		*Describe* vs. *REF* trial	*Match* vs. *REF* trial	

AD vs. HC	−	−0.70 (1.05, −0.34)	−	0.04; 0.21
MCI vs. HC	−	−	−
∣rule
	**Turn duration**	Turn duration * trial		

		*Describe* vs. *REF* Trial	*Match* vs. *REF* Trial	

AD vs. HC	−	−	−	0.07; 0.12
MCI vs. HC	−	0.33 (0.05, 0.61)	−0.27 (−0.53, −0.01)
∣rule
	**IPU duration**	IPU duration * trial		

		*Describe* vs. *REF* Trial	*Match* vs. *REF* Trial	

AD vs. HC	−0.28 (−0.38, −0.06)	−	−	0.04; 0.10
MCI vs. HC	−	−	−
∣rule
	**Tov duration**	Tov duration * trial		

		*Describe* vs. *REF* Trial	*Match* vs. *REF* Trial	

AD vs. HC	−	−0.96 (−1.63, −0.24)	−	0.04; 0.10
MCI vs. HC	−	−	−
∣rule
	**Gap/Overlap ratio**	Gap/Overlap ratio * trial		

		*Describe* vs. *REF* Trial	*Match* vs. *REF* Trial	

AD vs. HC	−	−	−	0.18; 0.42
MCI vs. HC	0.54 (0.19, 0.89)	−	−

*The exact p-value is given in the text. AD, Alzheimer’s disease; MCI, mild cognitive impairment; HC, healthy controls; *Describe*-Trial, individuals with AD/MCI and HC are the directors, i.e., they describe the shapes and instruct where to place them; *Match*-Trial, individuals with AD/MCI and HC are the matchers, i.e., they place the pictures on their board following the caregiver’s instructions; *REF-*Trial (*Describe and Match*-Trial), both interlocutors describe the shapes and agree on where to place them; N, number of; tov, transition overlaps; IPU, interpausal unit*.

**Table 4:** Coefficients and 95% confidence intervals with marginal and conditional *R*^2^ for the observed significant associations between the speech features and the cognitive domains (composite scores; *p* < 0.006).

Groups	Speech features	Speech features * trials		*R*^2^m; *R*^2^c
	Speech rate	Speech rate * trial	
		*Describe* vs. *REF* trial	*Match* vs. *REF* trial

Memory	−	0.26 (0.05, 0.47)	−	0.04; 0.21
Visuoconst./EF	−	−	0.28 (0.08, 0.48)
∣rule
	**Turn duration**	Turn duration * trial	

		*Describe* vs. *REF* trial	*Match* vs. *REF* trial

Memory	−	−	0.35 (0.16, 0.53)	0.06; 0.16
Visuoconst./EF	−	0.33 (0.17, 0.49)	0.18 (0.09, 0.14)
WM/Attention	0.40 (0.20, 0.59)	−0.36 (−0.56, −0.16)	−0.40 (−0.59, −0.21)
Orientation	−	−0.11 (−0.19, −0.02)	−

*The exact p-value is given in the text. *Describe*-Trial, individuals with AD/MCI and HC are the directors, i.e., they describe the shapes and instruct where to place them; *Match*-Trial, individuals with AD/MCI and HC are the matchers, i.e., they place the pictures on their board following the caregiver’s instructions; *REF-*Trial (*Describe and Match*-Trial), both interlocutors describe the shapes and agree on where to place them; Visuoconst./EF, visuoconstruction/executive function; WM/Attention, working memory/attention; N, number of; tov, transition overlaps; IPU, interpausal unit*.

**Table 5:** Coefficients and 95% confidence intervals with marginal and conditional *R*^2^ for the observed significant associations between the speech features and regional volumes in the fully adjusted models (per hemisphere; *p* < 0.003).

[-7pt]ROIs	Speech features	Speech features * trial		*R*^2^m; *R*^2^c
	Speech rate	Speech rate * trial	

		*Describe vs. REF trial*	*Match vs. REF trial*

L MTG	−	−	−0.32 (−0.51, −0.14)	0.04; 0.22
R STG	−	−	0.30 (0.13, 0.47)	0.04; 0.22
R MTG	−	−	−0.43 (−0.60, −0.25)
∣rule
	**Turn duration**	Turn duration * trial	

		*Describe vs. REF trial*	*Match vs. REF trial*

L MTG	−0.37 (−0.54, −0.20)	−	0.55 (0.39, 0.70)	0.09; 0.13
R MFG	−	0.14 (0.02, 0.25)	0.16 (0.05, 0.28)	0.07; 0.13
R STG	−	−0.17 (−0.29, −0.05)	−0.13 (−0.23, −0.01)
∣rule
	**Interpausal unit duration**	Interpausal unit duration * trial		

		*Describe vs. REF trial*	*Match vs. REF trial*

L MTG	−	0.16 (0.00, 0.23)	0.28 (0.21, 0.36)	0.04; 0.12
L ITG	0.15 (0.03, 0.27)	−0.11 (−0.16, −0.04)	−0.23 (−0.30, −0.16)
R IFG	−	−0.07 (−0.13, −0.02)	−0.10 (−0.16, −0.00)	0.04; 0.11
∣rule
	**Gap duration**	Gap duration * trial		

		*Describe vs. REF trial*	*Match vs. REF trial*	

R ITG	−	−0.02 (−0.3, −0.00)	−0.02 (−0.03, −0.00)	0.05; 0.18

*The exact p-value is given in the text. L, left; R, right; Cereb, cerebellum; FFG, fusiform gyrus; IFG, inferior frontal gyrus; IPL, inferior parietal lobule; ITG, inferior temporal gyrus; MFG, middle frontal gyrus; MTG, middle temporal gyrus; Prec, precuneus; STG, superior temporal gyrus; *Describe*-Trial, individuals with AD/MCI and HC are the directors, i.e., they describe the shapes and instruct where to place them; *Match*-Trial, individuals with AD/MCI and HC are the matchers, i.e., they place the pictures on their board following the caregiver’s instructions; *REF-*Trial (*Describe and Match*-Trial), both interlocutors describe the shapes and agree on where to place them; N, number of; tov, transition overlaps; IPU, interpausal unit*.

**Table 6:** Cross-validated accuracy rates of the evolved classifiers for the test set based on temporal speech features for the MCI-HC and AD-HC contrasts and for the multi-class classification of the three groups for the three trials separately.

	AD/HC % (SD)	MC/HC % (SD)	Multi-class % (SD)
*Describe*-trial	73.77 (9.65)	62.71 (4.78)	83.95 (3.32)
*Match*-trial	63.67 (3.43)	63.41 (5.91)	82.47 (2.20)
*Describe and match*-trial	70.79 (9.50)	62.50 (7.67)	84.17 (2.72)

*AD, Alzheimer’s disease; MCI, mild cognitive impairment; HC, healthy controls; *Describe*-Trial, individuals with AD/MCI and HC are the directors, i.e., they describe the shapes and instruct where to place them; *Match*-Trial, individuals with AD/MCI and HC are the matchers, i.e., they place the pictures on their board following the caregiver’s instructions; *Describe-Match*-Trial, both interlocutors describe the shapes and agree on where to place them*.

## Results

### Sample Characteristics

Table 1 provides descriptive statistics per group and the results from the least-square and generalized regressions. There was no statistical difference in age, gender or education level between the HC and the MCI or AD groups. Individuals with MCI and AD had lower RBANS and MOCA global scores compared to the HC (reference level). The Memory, Language, Working Memory/Attention, Visuoconstructional/Executive Function and Orientation composite scores were significantly lower for the AD group. MCI participants had lower Memory, Working Memory/Attention and Visuoconstructional/Executive Function composite scores. The AD group also had reduced Total Gray Matter volume.

### Speech Timing by Group

Table 2 provides the mean and standard deviation of the speech characteristics per Group × Trial. Significant results (*p* < 0.006, i.e., after Bonferroni correction) and tendencies or marginally significant results (*p* < 0.01, i.e., after Bonferroni correction) are reported herein. Significant coefficients, 95% confidence intervals and *R*^2^ are given for the three trials and per Trial when the interaction Group × Trial was significant (*p* < 0.006) in Table 3.

The interaction Group × Trial was significant for speech rate ($\chi_{\left(14\right)}^{2}$ = 19.15, *p* < 0.006), interpausal unit duration ($\chi_{\left(14\right)}^{2}$ = 21.38, *p* < 0.006) and turn duration ($\chi_{\left(14\right)}^{2}$ = 17.69, *p* < 0.006).

Individuals with AD had significant slower speech rate in the *Describe*-Trial compared to HC (*p* < 0.006). They also produced shorter interpausal units (*p* = 0.003) across the three trials. Their transition overlaps tended to be shorter in the *Describe*-Trial (*p* = 0.008). MCI participants tended to produce longer turns in the *Describe*-Trial (*p* = 0.01). The gap/transition ratio tended to be larger for the MCI groups (*p* = 0.009) compared to the HC across the three trials, i.e., individuals with MCI used more often a gap than a transition overlap when taking a turn. There was no significant (or marginally significant) difference in the number of pauses between the AC or MCI and HC groups.

Together, these results suggest that AD participants speak more slowly and take a longer time to respond when engaged in a collaborative referential task compared to HC. Our findings also suggest that MCI participants tend to produce longer turns. Their response times to take turns also tended to be longer than HC.

### Speech Timing—Domain-Specific Cognitive Function Association

Significant results (*p* < 0.006, i.e., after Bonferroni correction) and tendencies or marginally significant results (*p* < 0.01, i.e., after Bonferroni correction) are reported herein. Significant coefficients, 95% confidence intervals and *R*^2^ are given for the three trials and per Trial when the interaction Group × Trial was significant (*p* < 0.006) in Table 4.

#### Speech Rate

The interaction Group × Trial was significant for the Memory ($\chi_{\left(11\right)}^{2}$ = 18.53; *p* < 0.006) and Visuoconstructional/Executive function ($\chi_{\left(11\right)}^{2}$ = 19.22; *p* < 0.006) components. Slower speech rate was significantly associated with lower Memory scores in the *Describe*-Trial (*p* = 0.001) and with lower Visuoconstructional/Executive function scores in the *Match*-Trial (*p* = 0.004).

#### Turn Duration

The interaction Group × Trial was significant for the Working Memory/Attention ($\chi_{\left(11\right)}^{2}$ = 19.22; *p* < 0.006), Memory ($\chi_{\left(11\right)}^{2}$ = 18.53; *p* < 0.006) and Visuoconstructional/Executive function components ($\chi_{\left(11\right)}^{2}$ = 14.57; *p* < 0.006). Shorter turns were associated with lower Working Memory/Attention scores across the three trials (*p* < 0.006), with weaker associations for the *Describe*-Trial (*p* < 0.006) and in the *Match*-Trial (*p* < 0.006). Positive associations were also found with Memory scores in the *Match*-Trial (*p* < 0.006) and with Visuoconstructional/Executive function scores in the *Describe*-Trial (*p* < 0.006) and the *Match*-Trial (*marginal, p* = 0.01).

#### Interpausal Unit Duration

**Figure 3:**
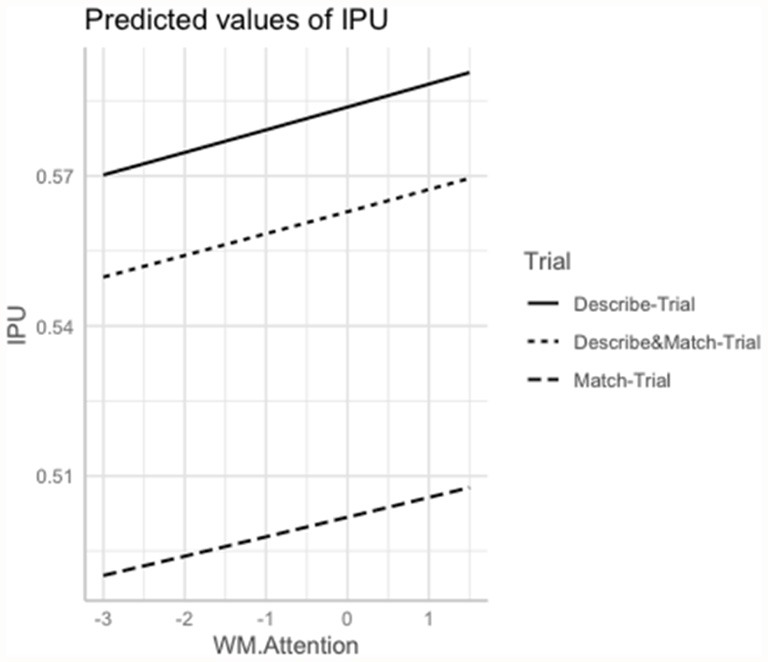
Marginal estimates of Interpausal Unit (IPU) duration (log-transformed) as a function of Working Memory/Attention scores (log-transformed) in the *Describe*, *Match*, and *Describe and Match* trials. *Describe*-Trial: individuals with AD/MCI and HC are the directors, i.e., they describe the shapes and instruct where to place them; *Match*-Trial: individuals with AD/MCI and HC are the matchers, i.e., they place the pictures on their board following the caregiver’s instructions; *Describe and Match-*Trial: both interlocutors describe the shapes and agree on where to place them.

The interaction Group × Trial was significant for Memory ($\chi_{\left(11\right)}^{2}$ = 11.13; *p* = 0.003) and marginally significant for Orientation ($\chi_{\left(11\right)}^{2}$ = 7.97; *p* = 0.01). Shorter interpausal units tended to be associated with lower Memory scores (*p* = 0.008) and with lower Orientation scores (*p* = 0.004) in the *Describe*-Trial. Interpausal units also tended to be shorter with lower Working Memory/Attention scores across the three trials (*p* = 0.009). See Figure 3.

#### Pause Duration

The interaction Group × Trial was marginally significant for the Orientation component ($\chi_{\left(11\right)}^{2}$ = 9.83, *p* = 0.007). Longer pauses tended to be associated with lower Orientation scores in the *Describe*-Trial (*p* = 0.01) and with lower Memory scores across the three trials (*p* = 0.004).

#### Gap/Transition Overlap Ratio

The interaction Group × Trial was marginally significant for the Language component ($\chi_{\left(11\right)}^{2}$ = 8.15; *p* = 0.01). A larger ratio (i.e., a higher occurrence of gaps as compared to transition overlaps) tended to be associated with lower Language scores in the *Match*-Trial (*p* = 0.006).

Gap duration, transition overlap duration and the number of pauses were not associated with any of the cognitive domains.

To summarize, slower speech rate and shorter turns were significantly associated with lower Memory and Visuoconstructional/Executive Function scores, with shorter turns being further associated with lower Working Memory/Attention scores. Marginal associations suggest similar trends. Shorter interpausal units tended to be associated with lower Memory and Working Memory/Attention scores as well as with lower Orientation scores. Lower Memory and Orientation scores tended to be associated with longer pauses.

### The Structural Correlates of Speech Timing

Significant results (*p* < 0.003, i.e., after Bonferroni correction) and tendencies or marginally significant results (*p* < 0.006, i.e., after Bonferroni correction) are reported herein. Significant coefficients, 95% confidence intervals and *R*^2^ are given for the three trials and per Trial when the interaction Group × Trial was significant (*p* < 0.003) in Table 5.

#### Speech Rate

The interaction ROI*Trial was significant for the L MTG ($\chi_{\left(12\right)}^{2}$ = 13.54; *p* < 0.003), R MTG ($\chi_{\left(12\right)}^{2}$ = 24.02; *p* < 0.003) and R STG ($\chi_{\left(12\right)}^{2}$ = 12.84; *p* < 0.003). In particular, slower speech rate was associated with smaller volume of L MTG and R MTG (*p* < 0.003) except in the *Match*-Trial; with smaller volume of R STG in the *Match*-Trial (*p* < 0.003).

#### Turn Duration

The interaction ROI*Trial was significant for the R MFG ($\chi_{\left(12\right)}^{2}$ = 10.36; *p* < 0.003), and the R STG ($\chi_{\left(12\right)}^{2}$ = 9.57; *p* = 0.003). Shorter turns were associated with smaller volumes of L MTG (*p* < 0.003) across the three trials. Shorter turns were also associated with smaller volume of R MFG (*p* = 0.003) in the *Match*-Trial and R STG (*p* = 0.003) in the *Describe*-Trial.

#### Interpausal Unit Duration

**Figure 4:**
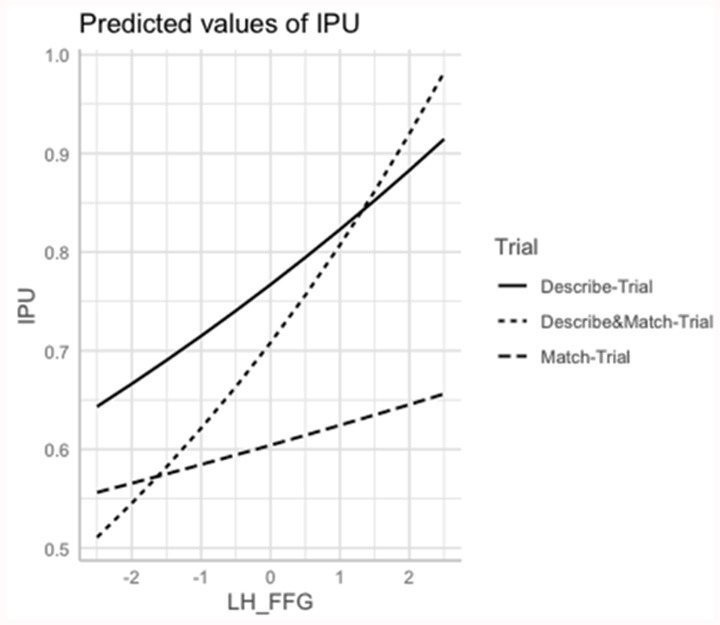
Marginal estimates of Interpausal Unit (IPU) duration (log-transformed) as a function of the Left Fusiform Gyrus (L FFG) in the *Describe*, *Match*, and *Describe and Match* trials. *Describe*-Trial: individuals with AD/MCI and HC are the directors, i.e., they describe the shapes and instruct where to place them; *Match*-Trial: individuals with AD/MCI and HC are the matchers, i.e., they place the pictures on their board following the caregiver’s instructions; *Describe and Match-*Trial: both interlocutors describe the shapes and agree on where to place them.

The interaction ROI*Trial was marginally significant for the L MTG ($\chi_{\left(12\right)}^{2}$ = 10.63; *p* = 0.004), L ITG ($\chi_{\left(12\right)}^{2}$ = 10.22; *p* = 0.006), and L Cereb ($\chi_{\left(12\right)}^{2}$ = 10.22; *p* = 0.007). Shorter interpausal units were associated with smaller volume of L MTG in the *Describe*- and *Match* Trials (*p* < 0.003), with smaller volume of L ITG in the *Describe*-Trial (*p* < 0.003) and with smaller volume of L Cereb in the *Describe*-Trial (*p* = 0.004). The R IFG volume was also positively associated with interpausal unit duration, with weaker association in the *Describe*-Trial (*p* = 0.003) and *Match*-Trials (*p* < 0.003). See Figure 4.

#### Gap Duration

Gap duration was positively associated with the R ITG volume except in the *Describe* and *Match*-Trials (*p* < 0.003).

No association between pause duration, transition overlap duration, number of pauses, gap/transition overlap ratio and the ROIs volumes were found.

To summarize, slower speech rate, shorter turns and shorter interpausal units were significantly associated with smaller volumes of L MTG. Speech rate was also positively associated with the R MTG and R STG volumes; turn duration with the R STG and R MFG; and interpausal unit with the L ITG, L IFG and L cereb. With regard to turn-taking organization, individuals with smaller R ITG volumes tended to produce longer gaps.

### MCI and AD Classification Based on Temporal Speech Measures

Table 6 presents the cross-validated accuracy rates of the evolved classifiers for the test set based on the temporal speech features for the MCI-HC and AD-HC contrasts and for the multi-class classification of the three groups for the three trials separately. Best performances were achieved for the classifiers that were based on the speech measures extracted from Trial 1 and Trial 3 for the AD-HC contrast. Accuracy rates were moderate in the pairwise contrasts and slightly better for the AD-HC contrasts compared to the MCI-HC contrasts (e.g., 73.77 vs. 62.71 in Trial 1). Accuracy rates were similar across trials for the multi-class classification of the three groups (82.47% to 84.17%).

## Discussion

In this preliminary study exploring the cognitive and structural underpinnings of temporal speech characteristics in a collaborative referential task, we first show that MCI and mild-to-moderate AD are characterized by a general slowness of speech, attributed to slower speech rate and slower turn-taking, with shorter transition overlaps and a larger number of gaps than transition overlap at speaker changes. Individuals with AD also had shorter interpausal units and individuals with MCI had longer turns. Our findings on speech rate, pauses and interpausal units corroborate other analyses of connected speech in MCI and AD (Singh et al., 2001; Hoffmann et al., 2010; Rousseaux et al., 2010; Gayraud et al., 2011; Ahmed et al., 2013; Pistono et al., 2016; De Looze et al., 2018). The temporal characteristics of turn-taking organization, with slower exchanges for MCI and AD, support the potential existence of underlying cognitive deficits related to speech planning difficulties. Gap durations were almost doubled in the AD group compared to the HC (1,230 vs. 690 ms). Given that it takes about 1,500 ms to plan a simple sentence (Griffin and Bock, 2000), it may be postulated that individuals with AD needed more time to simultaneously comprehend their interlocutor’s utterances, plan their answers and anticipate turn-endings.

More specifically, our analyses revealed that slower speech rate and longer pause duration were indicative of lower verbal memory scores and lower volumes of superior and middle temporal gyri. Slower speech rate was also associated with lower visuoconstructional/executive function scores and longer pauses with lower orientation scores.

Our findings suggest that slower speech rate and longer pause duration may be indicative of underlying deficits in episodic memory, lexical, semantic and executive functioning processes. Within the frame of the referential task, they may reflect difficulties with picture naming and remembering the sequence in which the pictures are described or remembering preceding exchanges (Feyereisen et al., 2007; Ash et al., 2011). Longer pauses may also reflect the time needed for the speaker to organize their thoughts and to construct a sentence. The associations observed with the superior and middle temporal gyri further support this interpretation. These regions have been linked to semantic memory and retrieval (Pravatà et al., 2016; Leyton et al., 2019) and to be dependent on an individual’s verbal working memory capacity (Deschamps et al., 2014). Our findings corroborate the associations observed in other studies between within-clause pauses and activation in the superior and middle temporal gyri bilaterally (Kircher et al., 2004) as well as between speech rate and the STG and the MTG.

In addition, shorter interpausal units and shorter turns were associated with lower memory and working memory/attention scores. Shorter interpausal units were further related to lower orientation scores. Within the frame of the referential task, it may be hypothesized that these characteristics may reflect the production of shorter sentences of simpler syntactic and discourse structure and/or may be indicative of a narrower scope of speech planning (Swets et al., 2013; De Looze et al., 2018). With regards to the structural correlates, shorter interpausal units were associated with volume reductions in the right IFG, the left middle and inferior temporal gyri and left cerebellum. Associations were also observed between shorter turns and lower volumes of left and right middle MTG and right STG. The IFG is thought to support lexico-semantic retrieval processes and the parsing and planning of sentence and discourse-level linguistic information (Hagoort, 2005; Binder et al., 2009; Matsumoto et al., 2013; Hurley et al., 2015; Foti and Roberts, 2016). More generally, it has been linked to executive function, working memory and attention (Tops and Boksem, 2011; Zheng et al., 2014; Nissim et al., 2017). The inferior and middle frontal regions and the STG have been linked to speech planning processes and timing control. Furthermore, the cerebellum has been associated with speech and language control, timing, anticipation/prediction during language comprehension, verbal working memory and mental manipulation (Stoodley and Schmahmann, 2009; Marvel and Desmond, 2010; Murdoch, 2010; Mariën et al., 2014). In a previous study (De Looze et al., 2018), using data from the whole cohort (*N* = 80), we showed that the same temporal speech features in overt sentence reading were associated with reduced working memory/attention and language scores. We suggested that the temporal speech features may not only be reflective of lexico-semantic deficits but also of speech production planning difficulties, potentially stemming from reduced working memory capacity and attention deficits, specifically in the context of increased cognitive-linguistic demand. Several studies have provided evidence that the scope of speech production planning (i.e., how far ahead speakers plan an upcoming utterance) varies both as a function of speaker-specific verbal working memory capacity and cognitive-linguistic demands (Rochon et al., 2000; Swets et al., 2007; Petrone et al., 2011). We postulate that the size of interpausal units and turns may result from reduced working memory capacity in a highly cognitively demanding task, underlying speech production planning difficulties with reduced scope of planning (De Looze et al., 2018). The associations observed with the right hemisphere for several of these regions may be reflective of the nature of the referential task, also relying on visuoconstructional and visuospatial skills when describing the geometrical shapes and when ordering and placing the pictures on the board (Baddeley, 2000).

Finally, our exploratory analyses showed moderate accuracy rates for the speech-based classifiers in the pairwise contrasts, with higher performance for the AD-HC contrast (74%) compared to the MCI-HC contrast (63%). The accuracy rates for the multi-class classification of the three groups (84%) were in line with other studies also using ensembles of acoustic features derived from picture-description tasks, interviews or a combination of different speech tasks (Singh et al., 2001; Roark et al., 2011; Jarrold et al., 2014; Meilán et al., 2014; Dodge et al., 2015; König et al., 2015; López-de-Ipiña et al., 2015; Asgari et al., 2017; Tóth et al., 2018; Gosztolya et al., 2019; O’Malley et al., 2020). Using a combination of linear and nonlinear acoustic features extracted from spontaneous speech samples, López-de-Ipiña et al. (2015) reported 87% accuracy for the discrimination of AD. Similar features extracted from several short cognitive tasks were also used for the classification of MCI and AD, reaching accuracies of 79% and 87% for the MCI-HC and AD-HC contrasts respectively (König et al., 2015). Using a set of acoustic features extracted from longitudinally collected biographic interviews and cognitive tests, Weiner et al. (2016) achieved a classification accuracy of 86% between HCs, individuals with aging-associated cognitive decline and individuals with AD. Other studies (Jarrold et al., 2014; Gosztolya et al., 2019) have combined acoustic and lexical or linguistic features derived from spontaneous speech and reported an accuracy of 86–88% for the AD-HC contrast and 80% for the MCI-HC contrast.

These findings together support the discriminative power of speech-based approaches and their clinical relevance as a diagnostic tool component for the assessment and monitoring of cognitive deficits in ageing. The advantage with speech-based approaches is that they are less computationally demanding, they can be fully automated, they are non-invasive, time and cost-effective and are easy to administer. For example, speech changes could be recorded and monitored using a mobile phone. Anonymized data could be sent and processed to the cloud and feedback about an individual’s cognitive functioning based on their speech characteristics, could be displayed and easily interpreted by a health professional *via* a web interface. Combining automated speech/language-based metrics with neuroimaging markers, neuropsychological scores and other behavioral measures, may assist health professionals in detecting and characterizing the course of cognitive decline in ageing and in defining an effective course of treatment and setting in place pertinent intervention strategies. These technologies may be of particular relevance in the context of stratification and screening procedures in overcrowded health services by providing some early insights (pending more in-depth clinical assessments) of an individual’s cognitive function and potential underlying structural changes.

A number of limitations need to be highlighted. First the sample size of this study was small, and restricted to a specific age, education and cognitive functioning groups, which limits the generalizability of our results. Second, we opted for a Region-of-interest (ROI) based approach which may have left out some existing associations not investigated in this study. This approach was chosen to exploit the richness of the repeated measures collected per individual through linear mixed-effect modelling. Finally, it is not possible from the present observational study to infer any direction of causality and the interpretations in this manuscript provided can but only be speculative, although supported by accumulated evidence stemming from an extensive literature review.

The novelty of this study lies in the investigation of the association between temporal speech parameters, cognitive domains and brain regional volumes in a collaborative referential task. Our study explores for the first time the use of automatically extracted conversational-based features as input of EAs for the classification of MCI and AD while, at the same time, provides a thorough description of the cognitive and structural correlates of these features, with the modest intention of bringing clinical evidence of the relevance of these behavioral measures for the assessment and monitoring of MCI and AD.

## Conclusion

Our study suggests that the temporal characteristics of speech in a collaborative referential task may reflect underlying cognitive deficits and structural volume reductions in healthy ageing, MCI and AD. The implementation of conversational speech-based technologies in clinical and community settings may represent a sensitive measure for the early assessment and longitudinal monitoring of cognitive-linguistic deficits and underlying structural changes in ageing.

## Data Availability Statement

The datasets presented in this article are not publicly available (following GPDR guidelines). Requests from research groups to access the datasets should be directed to deloozec@tcd.ie.

## Ethics Statement

The studies involving human participants were reviewed and approved by the St. James’s Hospital Ethics and Medical Research Committee. Signed informed consent was obtained from all respondents prior to participation. The patients/participants provided their written informed consent to participate in this study.

## Author Contributions

CD: drafting and stat analyses. AD: stat analyses and review. LC: data collection. AV: data annotation and review. RC, BL, and RR: input for data collection/analyses and review. All authors contributed to the article and approved the submitted version.

## Conflict of Interest

The authors declare that the research was conducted in the absence of any commercial or financial relationships that could be construed as a potential conflict of interest.

## Abbreviations

AD: Alzheimer’s disease
Cereb: cerebellum
CGP: cartesian genetic programming
EAs: evolutionary algorithms
FFG: fusiform gyrus
HC: healthy controls
IFG: inferior frontal gyrus
IPL: inferior parietal lobule
ITG: inferior temporal gyrus
L: left
MFG: middle frontal gyrus
MTG: middle temporal gyrus
MCI: mild cognitive impairment
Prec: precuneus
R: right
ROI: region-of-interest
STG: superior temporal gyrus.
